# Effect of combined training on the cardiorespiratory, muscle system and body composition in employees at a tertiary hospital after mild to moderate intensity COVID 19

**DOI:** 10.1016/j.clinsp.2025.100614

**Published:** 2025-04-25

**Authors:** Marcus Vinicius Grecco, Alexandre Moura dos Santos, JúliaMaria D'Andrea Greve, Angélica Castilho Alonso, Mara Silvia Afonso, Juliana Cristina de Sousa, Marília Simões Lopes Quintana, José Maria Soares-Junior, Edmund Chada Baracat, Guilherme Carlos Brech, Paulo Roberto Santos Silva

**Affiliations:** a Laboratory Study of Movement, Instituto de Ortopedia e Traumatologia do Hospital das Clínicas da Faculdade de Medicina da Universidade de São Paulo (IOT-HC-FMUSP), São Paulo, SP, Brazil; b Sports Medicine Division, Instituto de Ortopedia e Traumatologia do Hospital das Clínicas da Faculdade de Medicina da Universidade de São Paulo (IOT-HC-FMUSP), São Paulo, SP, Brazil; c FIFA Medical Centre of Excellence, São Paulo, SP, Brazil; d Department of Rheumatology at Faculdade de Medicina da Universidade de São Paulo (FMUSP), São Paulo, SP, Brazil; e Graduate Program in Aging Sciences, Universidade São Judas Tadeu (USJT), São Paulo, SP, Brazil; f Department of Obstetrics and Gynecology, Gynecology Discipline, Hospital das Clínicas da Faculdade de Medicina da Universidade de São Paulo (FMUSP), São Paulo, SP, Brazil

**Keywords:** combined training, COVID-19, Rehabilitation, Ergospirometry, Cardiopulmonary exercise testing

## Abstract

**Objective:**

To verify the effect of 12 weeks of Combined Training (CT) in patients suffering from COVID-19 of mild to moderate intensity.

**Methods:**

69 participants diagnosed with mild to moderate COVID-19 were evaluated, with shortness of breath as the main symptom. All of them were sent by the Centro de Atendimento ao Funcionário do Hospital das Clínicas da Faculdade de Medicina da Universidade de São Paulo (CEAC-HCFMUSP). The sample was divided into three groups: Group 1 (G1) – 26 patients diagnosed with COVID-19 evaluated before and after combined training together with nutritional guidance. Group 2 (G2) – 21 patients diagnosed with COVID-19 were evaluated before and after 12 weeks. Patients in G2 (control) did not participate in the protocol but were instructed to train outside the hospital and did not receive nutritional guidance. Group 3 (G3) – 22 patients diagnosed with COVID-19 were evaluated before and after combined training along with nutritional monitoring and post-workout whey protein supplementation throughout the duration of the protocol.

**Results:**

The three groups did not have significant statistical differences when compared to each other, but there was significant intragroup improvement in the analyzed variables. There was an improvement in cardiorespiratory, metabolic, and muscular fitness. Furthermore, late symptoms of the disease were reduced at the end of the program.

**Conclusion:**

The results of the dependent variables analyzed were significant within groups, CT was an efficient training model in improving the patient's general condition (fatigue and dyspnea) when training was controlled with supervision.

## Highlights

• COVID-19 and functional disability.
• combined training as a treatment for COVID-19 symptoms.
• Therapeutic approach to patients with COVID-19.

## Introduction

Systematic reviews have shown functional and psychological changes after physical training in post-COVID-19 patients.^1^ The study of combined training in patients with COVID-19 has been gaining international attention, with research and results from different countries contributing to understanding its benefits and applications.^1^^,^^2^

Therefore, justifying the effect of combined training in post-COVID-19 patients, the present study will contribute to the construction of a solid and relevant body of knowledge for the health area.^1^^,^^2^ Some review studies have shown the effectiveness of this training model in COVID-19.^3^ Research on combined training, especially in the post-COVID-19 context, still presents several gaps to be explored. Some of the least investigated areas include.

Emerging evidence shows improvement in lung function in several countries, such as China, Italy, and the United States, demonstrating that combined training can significantly improve lung function in patients with COVID-19, reducing dyspnea and the need for supplemental oxygen.^3^, ^4^, ^5^ International research proves that combined training helps individuals recover from fatigue and muscle weakness, common symptoms in post-COVID-19 patients, allowing them to resume their daily activities more easily. Ongoing studies are investigating the potential of combined training in preventing long-term complications from COVID-19, such as cardiovascular, pulmonary and musculoskeletal diseases.^3^, ^4^, ^5^, ^6^ The COVID-19 pandemic has brought with it a series of challenges to global health.^4^, ^5^, ^6^ Among the sequelae of the disease, muscle weakness and reduced cardiorespiratory capacity are frequently reported. In this context, physical exercise has emerged as a promising strategy for the recovery and rehabilitation of these patients.^6^ Preliminary results are promising, suggesting that regular exercise may be crucial in promoting post-COVID-19 health. Healthcare professionals worldwide are adapting to integrate combined training into recovery plans for COVID-19 patients, considering their physical conditions and individual needs. Therefore, the construction of more effective and individualized rehabilitation protocols like the one in this study is being developed based on the results of international research, optimizing the recovery process for patients with COVID-19.^1^^,^^2^^,^^4^, ^5^, ^6^ In this sense, international health bodies, such as the World Health Organization (WHO), are evaluating the evidence on combined training and considering the inclusion of recommendations in their guidelines for the management of COVID-19. International research on combined training in COVID-19 has offered promising results that demonstrate its benefits in recovering patients and preventing complications.^5^^,^^6^ Therefore, with continued studies and strategic implementation, combined training has the potential to become a valuable tool for managing COVID-19.^1^^,^^2^^,^^5^^,^^6^ These reviews have shown that training programs consisting of aerobic and resistance exercises can improve functional capacity and quality of life.^1^ Therefore, there is irrefutable evidence of the beneficial role of physical exercise in preventing diseases, as well as treating adjuvant in chronic diseases and psychological well-being. Furthermore, exercise may also have a protective effect on the immune system, the optimal state of which is crucial to adequately respond to the threat of COVID-19.^1^^,^^2^ This is especially important in chronic patients, who are affected by COVID-19. Studies on pulmonary rehabilitation using respiratory training, coughing exercises, diaphragmatic training, and stretching exercises observed significant improvements.^3^^,^^4^ In a randomized clinical trial, another study in patients with COVID-19 demonstrated that inspiratory muscle training improved symptoms, respiratory muscle strength, and aerobic fitness.^4^^,^^5^

The European Society of Cardiology, based on several pulmonary and cardiological changes that can be caused in patients with COVID-19, warns that not all published studies evaluated physical activity prescriptions safely and exercise prescriptions were considered only generic.^5^^,^^6^

However, there is evidence that exercise can protect the host from many other viral infections, including influenza, rhinovirus (another cause of the common cold), and herpes viruses such as Epstein-Barr (EBV), Varicella-Zoster (VZV), and Herpes Simplex Virus-1 (HSV-1). Therefore, maintaining physical activity levels is essential to reduce sedentary behavior, as this has a well-known detrimental effect on cardiorespiratory, metabolic, and muscular function with a clinically significant drop in the quality of life of many individuals.^3^, ^4^, ^5^, ^6^, ^7^

In a telephone survey of symptomatic adults who had a positive outpatient test result for SARS-CoV-2, 35 % had not returned to their normal state of health when interviewed 2 to 3 weeks after testing.^6^^,^^7^ Although conclusive validations in these patients present a certain bias, randomized clinical trials are necessary and, because of this uncertainty, the authors built a controlled Combined Training (CT) model based on the data available for patients with COVID-19. CT, by combining two physical strengths (aerobic endurance and strength), is a powerful tool to assist in the recovery and improvement of the quality of life of individuals who have physical weaknesses, and the authors believe that patients who have had COVID-19 will be able to benefit from this training model. Therefore, through a combination of aerobic and strength exercises, CT offers several physical and mental benefits, helping patients to feel healthier, more energetic, and more confident. This training modality is recommended for its positive adaptations in neuromuscular, cardiorespiratory, and metabolic aspects.^7^, ^8^, ^9^, ^10^ Therefore, the objective of this study was to show that just 12 weeks of a CT program is enough to improve indicators of functional fitness (aerobic capacity, strength, and body composition) in a population of patients with mild to moderate intensity COVID-19 without a history of hospitalization.

## Methods

### Study location and ethical issues

The study was performed in the Motion Study Laboratory of the Institute of Orthopedics and Traumatology University of São Paulo. The Ethics Committee for the Analysis of Research Projects case CAAE (39115320.9.0000.0068) approved the study. Registered at clinicaltrials.gov.br/rg/RBR-4r29z32. All signed a free and informed ethical consent.

### Type of study and participants

It was a prospective, controlled, and comparative study. The trial was conducted between August 2020 and September 2022 at the Institute of Orthopedics and Traumatology, Hospital of the Clínics, Faculty of Medicine, University of São Paulo.

### Sample

The study included 69 participants diagnosed with COVID-19, of mild to moderate intensity, with shortness of breath as the main symptom. However, 10 % (n = 7) had diabetes, 24 % (n = 17) systemic arterial hypertension, 44 % (n = 30) persistent headache, 46 % (n = 32) loss of smell, 49 % (n = 34) loss of taste, and 78 % (n = 54) had other symptoms (memory loss, hair loss, fatigue, etc.). All were referred by the Employee Care Center of the Hospital das Clínicas of the Faculty of Medicine of the University of São Paulo (CEAC-HCFMUSP). The sample was divided into three groups: Group 1 (G1) – 26 patients diagnosed with COVID-19 evaluated before and after combined training (12 weeks) together with nutritional guidance. Group 2 (G2) – 21 patients diagnosed with COVID-19 were evaluated before and after 12 weeks. G2 patients did not participate in the experimental protocol but were instructed to train outside the hospital and did not receive nutritional guidance. Group 3 (G3) – 22 patients diagnosed with COVID-19 evaluated before and after combined training along with nutritional monitoring and post-workout whey protein supplementation throughout the protocol (12-weeks). The randomization of patients was done by drawing lots. Allocation was concealed from all participants using sealed, sequentially numbered envelopes. Thus, 30 G1 labels, 30 G2 labels, and 30 G3 labels were allocated inside an envelope, drawn upon the patient's arrival. During the research process, the authors had a loss of 24 % of patients.

The inclusion criteria were: (i) Patients with COVID-19 without a history of hospitalization and with symptoms of shortness of breath, (ii) Aged between 18 and 60 years, (iii) Normal electrocardiogram, (iv) Absence of previous injury to any etiology in the last three months, (v) Absence of serious musculoskeletal disease, (vi) Absence of serious inflammatory, neurological, psychiatric disease and uncontrolled arterial hypertension, (vii) Not smoking and (viii) Being available to train in the Laboratory of Movement Studies (LEM). The exclusion criteria were: (i) Inability to perform any of the tests, (ii) Missing four consecutive training sessions, and (iii) Failure to attend the reevaluation tests.

### Clinical evaluation

Anamnesis (inclusion criteria), physical examination (blood pressure and bioimpedance). Blood pressure was measured in the sitting position with oscillometric devices according to the 2018 European Society of Cardiology/Hypertension guidelines. Arterial hypertension was defined as systolic BP ≥ 140 mmHg and/or diastolic ≥ 90 mmHg or using antihypertensive drugs.^11^ This criterion was used only for safety in combined training and not for study control. Bioimpedance was performed on the In Body 230 scale (In Body®, Gangnam-gu, Seoul, Korea), which verified the body mass index (kg/m^2^), percentage of fat/muscle (fat in kg/body weight), waist/hip ratio (cm), and rate metabolic (Kcal). A modified Medical Research Council dyspnea scale (mMRC Dyspnea Scale)^12^ was applied before and after the interventions in the three groups. The scale is made up of grades from 0 to 4, where 0 is feeling tired only during strenuous exercise and 4 is not having the energy to leave the house or get dressed. The questionnaire with a scale of 0 to 4 was applied at three different times: (1) Before COVID-19, (2) After COVID-19, and (3) After exercise training. All of these clinical assessment variables were control criteria during the protocol.

### Lower body strength assessment

Isokinetic knee strength was assessed to get a general idea of the patient's post-COVID-19 strength using the Biodex® Multi-joint System 3 isokinetic dynamometry (Biodex Medical TM, Shirley, NY, USA). The isokinetic dynamometer was calibrated 30 min before the start of the tests. Before the isokinetic test, patients underwent a 5-minute warm-up on a computerized cycle ergometer (Bike, Cardiovascular Systems, Cybex, USA). After a standardized warm-up, the subjects were positioned for concentric assessment of the knee joint extension and flexion movements. They remained seated with their hips flexed at 90° and secured to the chair using belts. The test started with the dominant limb. The limb was evaluated by positioning the lateral condyle of the femur in alignment with the mechanical axis of the dynamometer. All subjects performed four submaximal repetitions to familiarize themselves with the equipment, followed by a 60 s rest interval. They began the test at a determined speed of 60°/s (force) with 4 repetitions on each limb with a 1-minute break between them. After a brief rest, the test was performed at a speed of 240°/s (power) with 20 repetitions on each limb with a 1-minute break between them. Constant standardized verbal encouragement was given during testing to promote maximal effort during contractions. The isokinetic variables used were Peak Torque corrected for Body Weight at 60°/s (PTQ/BW) and total work (energy expended during the entire joint movement) at 240°/s (TW).^13^

### Cardiopulmonary exercise testing (CPX test)

Each subject underwent a graded exercise test at the Institute of Orthopedics, Movement Investigation Laboratory (IOT-HC-FMUSP). A modified version of the Heck stress test protocol was used with fixed speed and increasing slope increments at the rate of 2 % per minute. The speed was selected from those available (2.4, 3.6, 4.8, 6.0, 6.5, 7.2 km/h) based on the lack of individual conditions after two pilot tests with different speeds. Therefore, the protocol was self-chosen. Once chosen the speed individuals remained a minute at rest, and, soon after, started the protocol at the speed previously chosen and tested. Before testing, subjects were prepped for a 12-lead electrocardiogram and had standardized. All exercise tests were conducted on a motor-driven treadmill (h/p/cosmos™, pulsar, Nussdorf-Traunstein, Germany). Heart rate was recorded by a HeartWare electrocardiographic monitoring system (HeartWare™ Instruments, 6.4, Belo Horizonte, MG, BRA). During exercise, heart rate measurements were recorded during the last 10 s of every minute and at peak exercise by the electrocardiographic monitor. Blood pressure was determined via the auscultatory technique during the last minute of each stage and immediately after the test. Oxygen uptake (VO_2_) was measured continuously during the exercise test using an Ultima CPX™ Metabolic Stress Testing System (Medical Graphics™, Minneapolis, MN, USA). The instrument was calibrated with known volumes of O_2_ and CO_2_ and the pneumotach was calibrated with a syringe of known volume. Peak VO_2_ (VO_2_peak) was determined from the highest VO_2_ attained during the test. The perceived exertion was evaluated by the patient at each stage of the cardiopulmonary exercise test on a linear scale with 15 points (6‒20) as described by Borg. Three submaximal metabolic transition points were verified: (1) The Cardiorespiratory Optimal Point (COP) of minimum cardiovascular efficiency identified before VT1, (2) The Ventilatory Threshold 1 (VT1) considered the aerobic limit, and (3) The Ventilatory Threshold 2 (VT2) considered as respiratory compensation point. In addition, peak oxygen uptake (VO_2_peak). COP was determined by the lowest VE/VO_2_, the VT1 was determined when the Ventilatory Oxygen Equivalent (VE/VO_2_) and end-Expiratory Oxygen Pressure (PETO_2_) reached the lowest value, preceded by a continuous increase in both values, associated with an abrupt increase in the Respiratory Quotient (RQ = VCO_2_/VO_2_), called O_2_ set point, and VT2 was determined when the Ventilatory Equivalent of Carbon Dioxide (VE/VCO_2_) reached the lowest value preceded by its increase and the end-Expiratory Pressure of Carbon Dioxide (PETCO_2_) reached the highest value preceded by its reduction. VT2 is the second inflection of the curve in progressive exercise and the Respiratory Compensation Point (RCP) for VCO_2_ or known as the CO_2_ set point. The variables used for analysis were VO_2_peak (mL/kg/min), velocity (km/h), O_2_ pulse (mL/FC), work rate (Kgm) produced on the treadmill, maximum Heart Rate (HRmax), test duration (min). The loss of verbal communication with a subject during the test was overcome with prearranged hand signals. Measurement of peak Oxygen uptake (VO_2_peak) and other cardiorespiratory variables was repeated on each patient using an identical protocol after eight weeks of supervised physical training. The VO_2_max or VO_2_peak was taken as the highest VO_2_ value obtained in any 30 s period and was stated as being achieved by the following end-point criteria: (a) Oxygen uptake did not increase > 2.1 mL/kg/min (plateau in VO_2_) despite increasing workload, (b) With Respiratory Exchange Ratio (RER) values > 1.1, (c) Maximal HR within five beats/min of the age-predicted maximum by Tanaka equation (HRmax = 208 - [0.7*age]), (d) More than 18 on subjective Borg's scale.^14^

### Pulmonary function tests (PFT)

Spirometry is a physiological test that measures the maximal volume of air that an individual can inhale and exhale with maximal effort. All participants used the same equipment (CPX Ultima, MedGraphics®, St. Paul, MN, USA). Forced Vital Capacity (FVC), Forced Expiratory Volume in one second (FEV1), and FEV1/FVC ratio were measured during the test. PFT (pulmonary function test) data were collected as a percentage predicted based on previously published reference equations. FEV1/FVC was reported as the raw numerical ratio. The interpretation of the values obtained was based on the ATS-ERS criteria. The difference between Pulmonary Condition Function (PCF) values was determined and used in the analysis. All PFT data obtained is displayed as a predicted percentage and not as raw values.^15^ Percentages ≥ 70 % in the FEV1/FVC ratio are considered normal in your lung function. FEV1 is related to possible degrees of pulmonary obstruction and is considered normal with percentages ≥ 80 %. The authors apply the same concept when we analyze FVC, which is related to the degree of pulmonary restriction (≥ 80 %).

### Aerobic training design

All patients underwent two incremental exhaustive cardiopulmonary exercise test (CPX test) sessions, separated by a period of 12-weeks. The subjects in G2 performed the CPX test but were used as a control group. G1 and G3 participants underwent 12-weeks of a supervised concurrent physical training program on a treadmill. The training program was carried out twice a week and was supervised by a team of experts in assessing functional capacity and physical conditioning. The model used in the study was aerobic interval training lasting 30 min on an ergometric treadmill, following the steps: three minutes with low-intensity stimulus at the speed (Km/h) initially corresponding to the POC and after the patient's adaptation, the authors moved to VT1 (aerobic limit) where they remained at speed (Km/h) for two minutes as a high-intensity stimulus. The interval ratio was 2:3 (high intensity and low intensity). Then, with the improvement in cardiorespiratory resistance and if they were able to do so, the authors advanced to VT2 (limit of respiratory compensation point) as a high-intensity stimulus, alternating with the intensity of VT1 (aerobic limit) as low intensity. A heart rate monitor (Polar F1™) was used in the study for patient control and safety.^16^ Due to the low aerobic level of the studied group (shortness of breath), CT always started with aerobic exercise on a treadmill.

### Resistance exercises training

They were performed two times a week in the weight room located in the laboratory. The muscles were trained: (a) Knee extensors and flexors, (b) Pectorals, (c) Latissimusdorsi, (d) Lumbar extensors, (e) And abdominals. A Maximum Repetition test (1RM) was the strategy for determining the maximum achievable load. Three sets were performed (15 repetitions) with 60 % of 1RM in the upper limbs and lower limbs. Loads were adjusted every two weeks (10 %‒15 %) following the progression principle (American College of Sports Medicine).^17^

### Statistical analysis

Descriptive statistics. For quantitative variables, the authors utilized the mean and standard deviation, or median and interquartile ranges (Q1 = 25 %‒Q3 = 75 %), depending on the distribution. Qualitative variables were represented through absolute frequency (count) and relative frequency (percentage). Inferential statistics. To assess variations between interventions at distinct time points, the authors employed the Two-Way Repeated Measures Analysis of Variance (ANOVA) involving two criteria (factors) of repeated measures. Additionally, the authors conducted the Tukey post hoc test for multiple comparisons, which extended to non-parametric variables as well, owing to the test's robustness and the absence of a non-parametric counterpart. Similarly, the Tukey post hoc test (HSD) was employed for conducting multiple comparisons. The test selection was justified by referencing the Central Limit Theorem and its application due to the likely normal distribution of the population. The authors examined potential correlations using the Pearson correlation coefficient (*r*) or the Spearman rank correlation test (rho). The Chi-Square test (χ^2^) or Fisher's exact test was employed to assess associations between variables, contingent upon statistical assumptions. Finally, significance was considered at α < 0.05. The statistical analyses were performed using R v.4.3.2, in the Windows environment (R Core Team, Vienna, Austria).

## Results

The distinction between intergroup and intragroup is crucial to understanding the results of the present study. There was no difference among Groups 1, 2, and 3 (intergroup) in the distribution by gender, age, time of infection, Body Mass Index (BMI), pre-existing diseases (diabetes and hypertension) and post symptoms (headache, lack of taste and smell) in patients with COVID-19. The authors also found no difference when we analyzed other symptoms (memory loss, hair loss, generalized pain, etc.) (Table 1).

**Table 1:** Clinical and demographic characteristics of study participants.

	G1 (n = 26)	G2 (n = 21)	G3 (n = 22)	p
**Age**	47 ± 8.1	40 ± 9	46 ± 12	0.06
**BMI (kg/cm^2^)**	30 ± 5	31 ± 7	28 ± 5	0.30
**Men, n (%)**	5 (19)	6 (29)	7 (27)	0.70
**Women, n (%)**	21 (81)	15 (71)	16 (73)	0.70
**Time of infection (months)**	6 ± 2	5 ± 2	7.5 ± 4	0.20
**Comorbidities pre-COVID-19**				
Diabetes, n (%)	4 (15)	1 (5)	1 (4.5)	0.30
SAH, n (%)	10 (38)	2 (9.5)	5 (23)	0.07
Others, n (%)	4 (15)	4 (19)	2 (9)	0.60
**Sintoms pos-COVID-19**				
Headache, n (%)	14 (54)	7 (33)	10 (46)	0.40
Smell, n (%)	10 (39)	12 (57)	9 (41)	0.40
Taste, n (%)	10 (39)	13 (62)	10 (45)	0.30
Breathlessness, n (%)	26 (100)	21 (100)	22 (100)	0.70
Others, n (%)	21 (81)	16 (76)	17 (77)	0.90

Comorbidities pre-COVID-19: Grade 1 osteoarthritis, asymptomatic disc herniation, controlled asthma.

Sintoms pos-COVID-19: Memory loss, hair loss, generalized pain.

Group 1 (G1) participants diagnosed with COVID-19 evaluated before and after combined training (3-months of training) together with nutritional guidance. Group 2 (G2) participants diagnosed with COVID-19 were evaluated before and after three months. G2 patients were not monitored according to the protocol but were instructed to train outside the hospital and did not receive nutritional guidance. Group 3 (G3) participants diagnosed with COVID-19 were evaluated before and after combined training along with nutritional monitoring and post-training whey protein supplementation throughout the protocol (3-months).

**Fig. 1:**
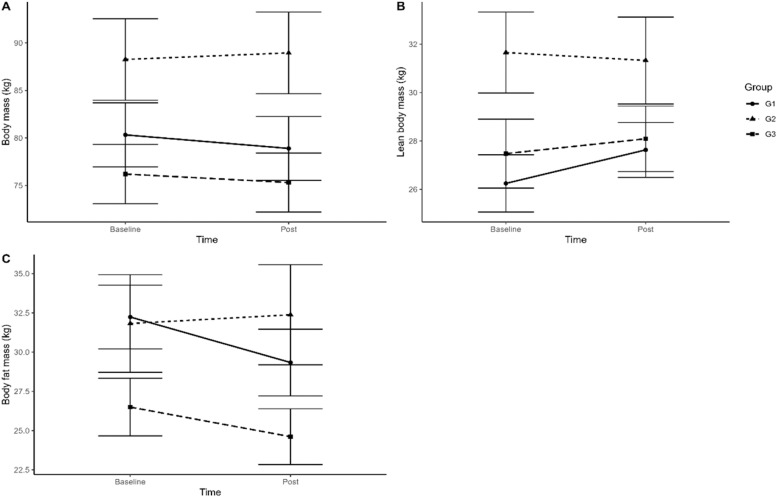
Comparison between body weight (A), lean mass (B) and fat mass (C) in pre and post training moment in groups G1, G2 and G3.

No statistical difference (p ≥ 0.05) was observed in the body composition of the variables analyzed: weight, fat mass, lean mass, BMI, % fat, waist/hip ratio, and intergroup basal metabolic rate. There was no statistical difference between the groups analyzed in body composition. However, in intragroups 1 and 3 (controlled training) there were better results than in group 2 (control group) as the authors can see in Fig. 1 representing body weight (A), lean mass (B), and fat mass (C).

**Fig. 2:**
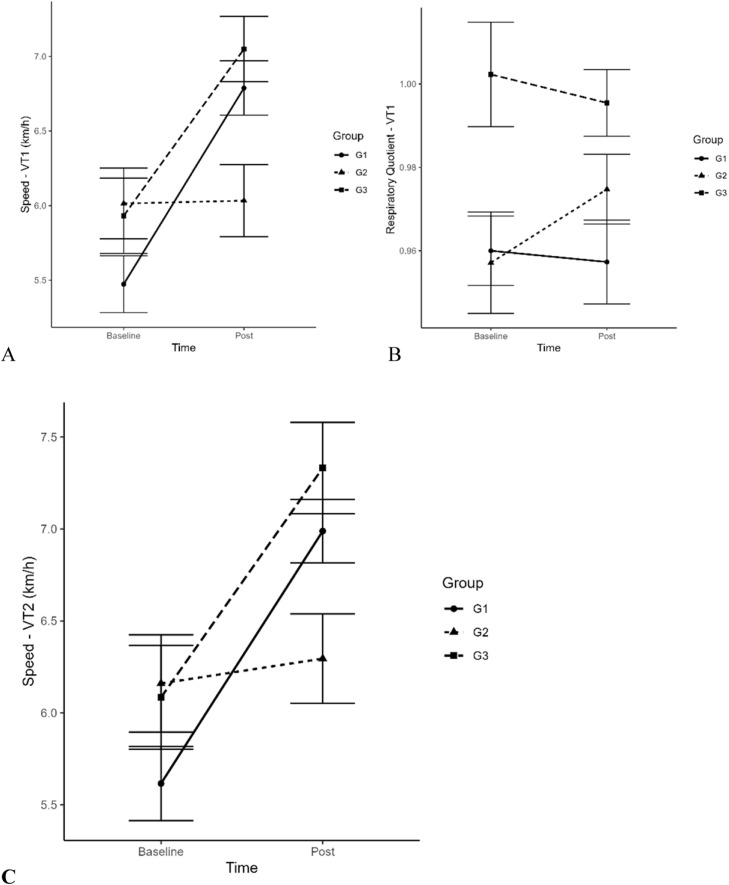
Comparison between speed and respiratory quotient in VT1 and VT2 in pre and post training moment in groups G1, G2 and G3.

**Fig. 3:**
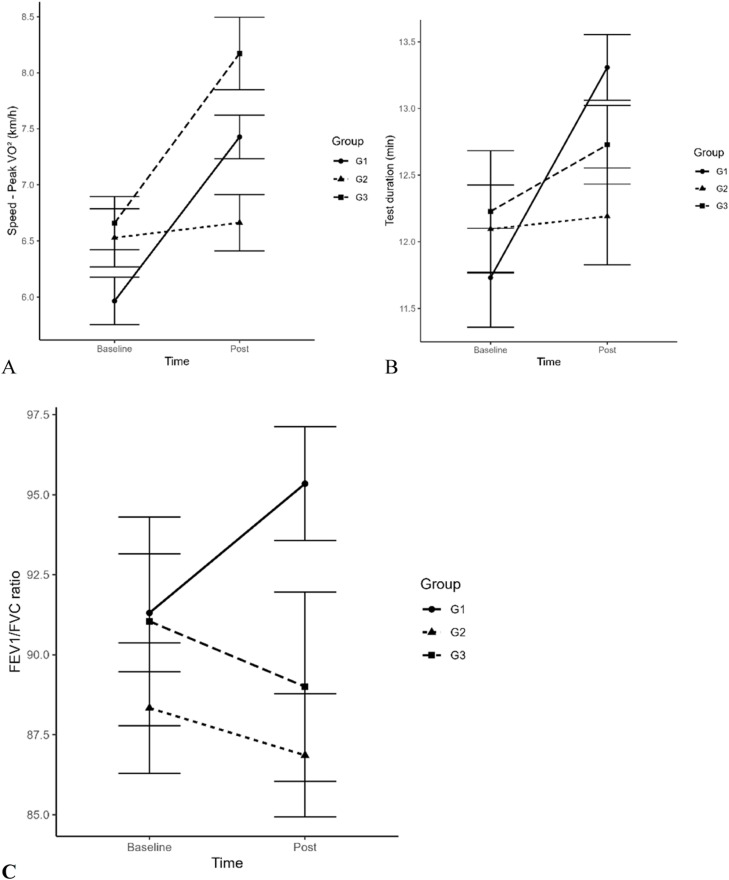
In the spirometry test the comparison between speed at peak VO_2_max, test duration and relation in FEV1/FVC in pre and post training moment in groups G1, G2 and G3.

In the cardiorespiratory assessment, the authors did not observe any difference between groups at the time of the COP, where the following parameters were verified: VO_2_, HR, speed, O_2_ pulse, and respiratory quotient (p ≥ 0.05). In VT1 the authors analyzed the same variables, adding only the variable work produced (kgm), we observed a significant difference (p = 0.002) only in the speed variable in G1 after the intervention and a significant difference (p = 0.0001) in G1 pre vs. G3 after the intervention (Fig. 2A). The variable Respiratory Quotient (RQ) in VT1 was significantly modified (p ≤ 0.05) in the intragroup analysis (Fig. 2B). In VT2 the authors analyzed all variables and verified differences in groups 1 and 3 (trained) only in the speed variable, where G1 (p = 0.0002) and G3 (p = 0.005) after training were significantly modified (Fig. 2C). At the VO_2_max peak, where the authors also analyzed all the variables presented at VT1 and VT2, we verified that the variable tolerance time to reach the two metabolic transition points and the peak velocity in the CPX test were significantly modified in G1 (p = 0.00023) and G3 (p = 0.005) after the intervention, respectively (Fig. 3A). Submaximal aerobic capacity was increased within G1 and G3, after training, respectively. In the test duration variable (exertion tolerance), a significant difference (p = 0.01) was verified in G1 after training (Fig. 3B).

In the spirometry test, the authors did not observe any difference in the variables analyzed (p ≥ 0.05): Reached Forced Vital Capacity (FVC), % FVC (related to sex, body weight, and age), Forced Expiratory Volume in the first minute reached (FEV1), %FEV1 (related to sex, body weight, and age), and FEV1/FVC ratio. This relationship can be seen in Fig. 3C.

**Fig. 4:**
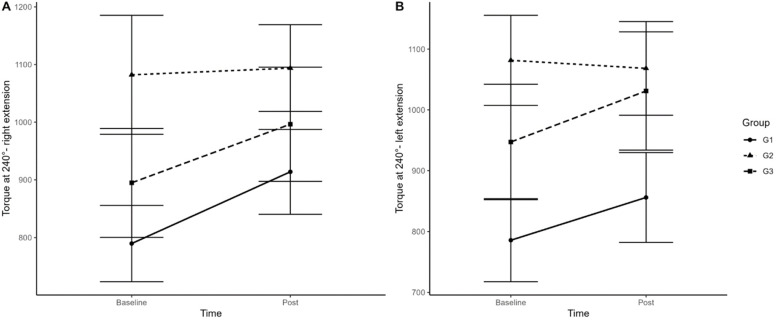
Isokinetic Peak of Torque in 240° knee extension in pre and post training moment in groups G1, G2 and G3.

In the isokinetic assessment, no statistical differences (p ≥ 0.05) were found between and within groups between the tests performed. Both the PTQ/PC ratio at 60°/s of the left/right knee extensor and flexor muscle group and the TT at 240°/s in the same joint and muscle group, with their respective deltas, the authors did not observe significant differences in the results. The authors can see this information in Fig. 4, which shows the TT 240°/s of the knee extensor muscle of the right (Fig. 4A)/left (Fig. 4B) lower limbs.

**Fig. 5:**
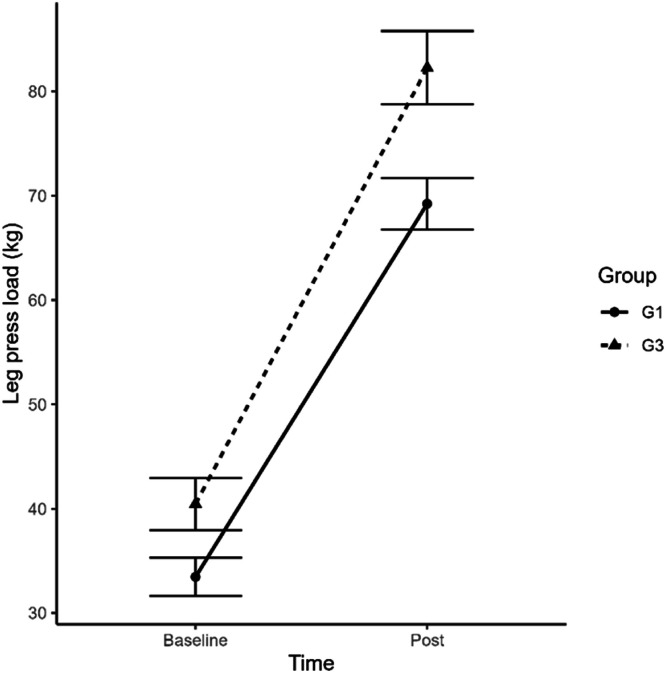
Load progression in the leg press exercise in pre and post training moments in groups G1 and G3.

Interval training carried out on a treadmill to improve cardiorespiratory and metabolic capacity, between POC and VT1 significantly modified the speeds of these two moments. Thus, in the POC speed after training, G1 (p = 0.00003) and G3 (p = 0.0001) showed a significant increase in performance, respectively. In strength training in the leg-press exercise, the authors also observed significant differences (p = 0.000006) in G1 and (p = 0.00002) in G3 in the increase in the load produced on the devices used during the training sessions. The G3 had a slightly greater increase in load in the leg-press exercise as we can see in Fig. 5. In the chest exercise after training, there was a significant increase in G1 (p = 0.0000001) and G3 (p = 0.00000000003), respectively. In the dorsal exercise, both trained groups had a significant increase, with G1 (p = 0.00000000002) and G3 after training (p = 0.00000000001), respectively. In the abdominal exercise, both had an increase in strength, with G1 (p = 0.002) and G3 (p = 0.004), respectively. In G3 who used a protein supplement, there was a slight advantage in strength gain over G1 (without supplement), but with no statistical difference between them.

**Fig. 6:**
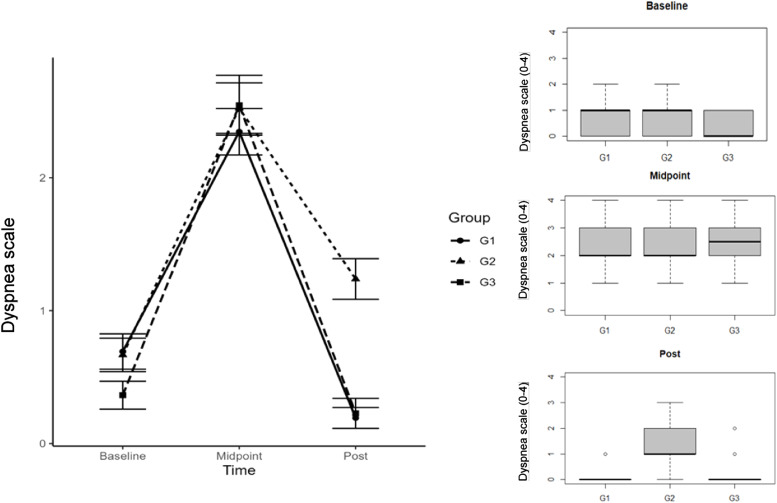
Dyspnea scale in pré, during and post CODI -19 in groups G1, G2 and G3.

On the dyspnea scale, when the authors compared the groups that trained under supervision (G1/G3) with G2 (control) there was a statistically significant difference (p = 0.0000001) (Fig. 6). However, overall, even without major statistical differences, controlled training (G1/G3) was more effective than uncontrolled training (G2).

## Discussion

The main result of this study is that the differences observed in intergroup variations (G1, G2, and G3) were not significant. However, when we compared the results within the trained groups (G1 and G3), there were significant changes in the studied variables that justified the engagement of these patients in CT. Therefore, the intragroup analysis shows that both groups (G1 and G3) showed significant improvement in almost all variables, regardless of exercise intensity. The statistical significance of the dependent variables in G1 and G3 (trained) indicates that the difference between the groups is unlikely to have occurred at random. In the present study, it suggests that the difference observed in G1 and G3 was relevant in the practice of CT.

As observed in the present study, some authors show that after COVID-19, the relationship between dyspnea, asthenia, and exercise intolerance stands out in these patients, justifying that a training program can have an impact on improving fatigue and dyspnea in these patients.^18^, ^19^, ^20^ Strength valence is a dependent variable in many health studies, including cardiorespiratory diseases since its effects are important for increasing muscular endurance and exercise tolerance.^21^ Some studies show that the effects of strength training are greater with durations greater than 12 weeks of training, with 30‒60 min per session and three sessions per week.^22^ However, it has been shown that just eight weeks of isolated strength training is enough to increase functional capacity.^22^, ^23^, ^24^ However, when dealing with frail individuals, other studies^24^, ^25^, ^26^ report that a minimum of physical activity per week (2×) can clinically change the patient by increasing strength, and aerobic capacity and improving body composition.^26^ These results are confirmed by Schoenfeld et al.^27^ who showed that the greatest effects of CT on strength are frequency-dependent on three weekly sessions. In the present study, the authors observed that the duration of 12 weeks of training and frequency of twice a week was not enough to achieve significant changes in body composition verified by the bioimpedance technique when we combined the three groups. However, in Groups 1 and 3 (supervised training), the authors observed a decrease in body weight of an average of 2 kg after intervention and Group 2 (control) showed no reduction at all. When we analyze the complete sample, the loss drops to 0.5 kg. The same happens when the authors quantify the gain in muscle mass, which increased by 0.5 kg in 12 weeks. However, when we verified the sample from groups 1 and 3, they gained an average of 1.2 kg while G2 (control) had no change in lean mass after the protocol was performed. Even though the changes were insignificant, controlled training was superior to G2 (control). These small changes as a result of TC training may be related to the similar training volume between exercise interventions.^28^^,^^29^

It is known that aerobic interval exercises improve VO_2_peak and training programs lasting eight to 12 weeks can increase VO_2_peak by more than 40 %.^30^^,^^31^ In the present study, the variable speed at the POC moment and VT1, submaximal condition, significantly improved by 30 % and 27 %, respectively. This result confirms that CT can contribute to a greater gain in the submaximal aerobic condition when compared to isolated continuous aerobic exercises.^9^ The values obtained in VO_2_peak in G1 and G3 (trained) ranged from 1 to 3 L/min respectively, while G2 obtained only 0.5 L/min after TC. Another variable that signaled performance was the peak speed in the CPX test, the G1 and G3 increased by almost 2 km/h while the G2 had no gain at all. Controlled and supervised training once again showed superiority when compared to the control group.^8^^,^^9^

The test duration increased, especially in G1, by almost 2 min and in G3 by 1 min, which means increased exercise tolerance. Strength training and its peripheral changes are not antagonistic to the development of aerobic capacity.^32^ On the contrary, the CT effect in this study improved cardiorespiratory aerobic strength and endurance and exercise tolerance.^32^, ^33^, ^34^ These studies help to demystify the idea that strength exercises harm aerobic capacity.^10^ Because of the results verified in the present study, the authors can suggest that strength training carried out in conjunction with endurance training can provide a significant increase in maximum strength, as reported by several authors.^35^

Another dependent variable that predicts physical capacity is muscular strength.^25^^,^^36^ It is known that the gain in muscle strength is largely responsible for functional changes in debilitated people.^30^^,^^36^, ^37^, ^38^ In the present study, the peak torque/weight ratio of the right and left knee extensor groups of G1 and G3 (trained groups) increased strength by 12 % and 8 %, respectively. The same response was seen in the total work of the knee extensor group on the right/left side, where G1 had an average gain of 12 % and G3 of 10 %, respectively. G2 (control) did not show significant gains in the variables studied by isokinetic dynamometry. This gain can be explained by a combination of interrelated factors. The training likely led to greater activation of quadriceps motor units, especially during eccentric and concentric knee.^39^ This means that more muscle fibers were recruited and worked together more efficiently to generate force. Furthermore, training may have improved the nervous system's ability to recruit fast-twitch muscle fibers, which generate more strength and power. We also cannot forget that repetitions of the exercises may have optimized the coordination between the quadriceps muscles and the stabilizing muscles of the knee joint, leading to a smoother and more efficient movement.^38^^,^^39^ It is known that quadriceps/hamstring strength training improves isokinetic peak torque at a speed of 60°/s in groups that present some frailty.^40^ In strength training sessions, a significant load gain was observed in the leg press exercise. Groups 1 and 3 (trained) exercised at 110 % and 130 %, respectively. Significant strength gains were also observed in other trained muscle groups but without significant statistical differences between them. The authors found that the group that had protein supplementation (G3, trained) did not obtain such significant results in strength and body composition when compared to the group without supplementation (G1, trained). According to several researchers, the main stimulus for hypertrophy and changes in body composition is training and nutritional re-education and not necessarily the consumption of supplements that promise miracles.^15^, ^16^, ^17^^,^^25^^,^^41^ In this sense, Schmitt et al.^42^ states that gaining muscle strength in the quadriceps is a preventative action and is essential to avoid knee injuries and improve walking or running performance during exertion. This statement is in line with what the authors found in the present study with greater speed gains on the treadmill and consequently gains in aerobic resistance in the studied patients.

Dyspnea was a common symptom across the entire study sample. When we compared G1 and G3 undergoing CT, the authors found a statistically significant difference (p = 0.0001) in the improvement of symptoms. On the contrary, in G2 (control) the symptoms remained. In pre-evaluation spirometry, no impairment of flows and volumes was found and, therefore, there was no restriction or obstruction to airflow. The dyspnea symptoms were likely related to the patient's low physical capacity.^8^^,^^9^ Although insignificant, after the CT protocol, G1 showed a 4 % gain in the Tiffeneau index while G2 and G3 showed 2 %, respectively. This response confirms that controlled physical activity can improve lung capacity and its corresponding benefits.^9^^,^^31^ Therefore, controlled CT sessions proved to be efficient in improving the cardiorespiratory quality of patients after COVID-19.^8^^,^^9^^,^^30^^,^^31^

In the present study, it was clear that groups 1 and 3 (trained) had an advantage in the results, especially when the authors saw an increase in aerobic resistance and strength. Carvalho et al.^25^ and Fisher et al.^26^ comments that 16 CT sessions are enough to notice functional changes in fragile people. The improvement in self-esteem was observed during the research but was not the subject of the study.^36^ It is important to highlight that only conventional physiotherapy sessions recruit less than 40 % of muscle fibers, generating weak stimuli when compared to controlled strength training that recruits above 60 %.^38^^,^^43^ The present results demonstrate that a concurrent exercise program combining aerobic and strength activities is highly effective in improving the physical fitness of patients recovering from COVID-19. The combination of aerobic and strength exercises appears to stimulate synergistic physiological adaptations, such as increased maximal oxygen consumption, muscle strength, and functional capacity. These benefits may be attributed to (explain the physiological mechanisms, e.g., increased mitochondrial biogenesis, muscle hypertrophy, improved endothelial function). These findings have important implications for clinical practice. Physical therapists and physical educators can use these results to develop more personalized and effective rehabilitation programs for patients with COVID-19. Furthermore, family physicians can recommend concurrent exercise as part of the multidisciplinary treatment for these patients.^25^^,^^26^^,^^36^^,^^43^^,^^44^, ^45^, ^46^, ^47^

### Limitations of the study

A limitation of the present study was the use of a small sample, which could generate less precision in the estimates, which means that there is a greater chance that the study results will not be an accurate reflection of reality with the limited possibility of generalizing the results to a broad population. However, in the individuals in the intragroup (G1 and G3) who underwent CT with supervision, the improvement of these individuals signaled the importance of this training model to improve the physical fitness of the participants. It is important to highlight that a small sample is not always a fatal limitation for a study. In some cases, studies with small samples can provide valuable information, especially when it comes to exploratory research or case studies. Although the present study was a short-term significant improvement in performance indicators, it is recommended that health professionals consider the inclusion of combined training in the rehabilitation programs of these patients, individualizing the program according to the needs and tolerance of each individual.

### Practical Implications and Conclusions

The study of concurrent physical training in patients with COVID-19 offered valuable insights for several sectors, with practical implications that transcended the scientific scope. Here, the authors explore the significance of this research for society, healthcare professionals, the healthcare system and other relevant areas. Physical training can help in the recovery of post-COVID-19 patients, reducing fatigue, muscle weakness and dyspnea. This contributed decisively to a better quality of life, allowing individuals to resume their routine activities more easily. Furthermore, regular physical training can help prevent long-term complications, such as cardiovascular, pulmonary and musculoskeletal diseases. This has benefited individual health and lessened the burden on the healthcare system. Therefore, the study encouraged patients to adopt an active lifestyle, with greater awareness of the value of chronic exercise, even after recovery from COVID-19. This promoted the general health and well-being of the trained population, reducing the risk of several chronic diseases. The results of the study allowed physiotherapists, physical education teachers, doctors and other professionals in the field to have a view of welcoming these patients with COVID-19 on the safe and effective practice of physical exercise during recovery. Another broadly positive aspect was the consecration of concurrent exercise (aerobic + strength) which endorsed the protocol developed at the Movement Studies Laboratory at IOT-HCFMUSP for the development of more individualized and effective rehabilitation for patients with COVID-19, taking into account consideration of your physical conditions and specific needs. It was clear that the benefits and risks of concurrent physical training in these patients allowed professionals in the field to make more informed decisions and offer superior quality care. Physical training reduces the need for hospital admissions for patients and reduces the burden on the healthcare system and optimizes the medical resources used. The study once again accelerated and reinforced the importance of physical activity in preventing diseases, including COVID-19. This response can be applied to other comorbidities and lead to a general reduction in healthcare costs and an improvement in the population's quality of life.

## Authors’ contributions

Marcus Vinicius Grecco: Investigation and Writing-original draft and Supporting.

Alexandre Moura dos Santos: Supporting Investigation and Writing-review & editing.

Júlia Maria D'Andrea Greve: Supervisor and Writing-review & editing.

Angélica Castilho Alonso: Supporting Investigation and Writing-review & editing.

Mara Silvia Afonso: Supporting Investigation and Writing-original draft.

Juliana Cristina de Sousa: Supporting Investigation and Writing-original draft.

Marília Simões Lopes Quintana: Supporting Investigation and Writing-original draft.

José Maria Soares-Junior: Supporting Investigation and Writing-review & editing.

Edmund Chada Baracat: Supporting investigation and writing-review & editing.

Guilherme Carlos Brech: Supporting investigation and writing-review & editing.

Paulo Roberto Santos Silva: Investigation and writing-review & editing.

## Declaration of competing interest

The authors declare no conflicts of interest.
